# The causal relationship between obstructive sleep apnea and rheumatic disease: A bidirectional Mendelian randomization study

**DOI:** 10.1515/rir-2025-0005

**Published:** 2025-04-02

**Authors:** Ming Chen, Heng Cao

**Affiliations:** Department of Rheumatology & Immunology, the First Affiliated Hospital, Zhejiang University School of Medicine, Hangzhou 310003, Zhejiang Province, China

**Keywords:** obstructive sleep apnea, rheumatic disease, mendelian randomization, causal relationship

## Abstract

**Background and Objective:**

Multiple studies have shown a substantial association between obstructive sleep apnea (OSA) and rheumatic disease. However, traditional studies are susceptible to confounding factors or reverse causal relationships, and the exact causal relationship still needs to be clearly defined. This study aims to use a bidirectional two-sample Mendelian randomization (MR) analysis to investigate the causal association between OSA and rheumatoid immune diseases.

**Methods:**

We conducted a two-sample bidirectional MR analysis by using large-scale genome-wide association studies (GWAS) summary statistics to investigate whether there is a causal relationship between OSA and rheumatic disease. Inverse variance weighted (IVW) was used as the primary analysis approach, supplemented by MR-Egger and Weighted median methods. Sensitivity analyses were conducted to ensure the robustness of the results.

**Results:**

The MR predicted ankylosing spondylitis (AS) was associated with risk of OSA (IVW: OR = 1.0239, 95% CI = 1.0086 to 1.0394, *P* = 0.0021; MR-Egger: OR = 1.0374, 95% CI = 1.0089 to 1.0668, *P* = 0.0326; weighted median: OR = 1.0287, 95% CI = 1.0109 to 1.0467, *P* = 0.0014). However, no bidirectional causal association was found between other rheumatic disease and OSA. The sensitivity analysis confirmed the robustness of the results.

**Conclusions:**

Our analysis suggests a potential causal relationship between AS and OSA. There was no direct causal relationship between OSA and other rheumatic disease. We need more experimental research on specific pathological and physiological mechanisms in the future.

## Introduction

Obstructive sleep apnea (OSA) is a prevalent and severe sleep disorder, with a prevalence rate of up to 9%-38% among the total population.^[1]^ OSA is a sleep disorder characterized by repeated, partial, or complete blockage of the upper airway during sleep, leading to reduced (hypopnea) or absent (apnea) airflow lasting at least 10 seconds, often accompanied by either cortical arousal or decreased blood oxygen levels. Common symptoms of OSA include intermittent hypoxia, hypoxemia, and disrupted sleep patterns.^[2]^ Furthermore, intermittent hypoxia can stimulate the release of inflammatory markers such as tumor necrosis factor-κB, interleukin (IL)-6, and IL-1β, which can provoke a systemic inflammatory response.^[3]^ It can be inferred that OSA is related to systemic inflammation and involves the activation of various cytokines. Moreover, the immune regulation pathway is a complex network that relies on mutually balanced interactions between cytokines and the cellular system.^[4]^ Rheumatic disease are systemic diseases, with their pathological basis being autoimmune inflammation and immune damage. The characteristic of rheumatic disease is immune system dysfunction, which activates immune cells to attack their antigens, leading to inappropriate inflammation and multi-tissue damage. Given this background, the relationship between rheumatic disease and OSA is noteworthy.

Several cohort studies indicate that patients with OSA are at a higher risk of developing rheumatic disease compared to controls. These diseases include rheumatoid arthritis (RA), Sjogren’s syndrome (SS), Behcet’s disease (BD), gout, and psoriatic arthritis (PsA), while the risk of systemic lupus erythematosus (SLE) and systemic sclerosis (SSc) appears less significant.^[5, 6, 7, 8]^ In addition, an increased prevalence of OSA has been observed in patients with various rheumatic disease, including RA, SS, BD, SLE, gout, PsA, SSc and anky-losing spondylitis (AS).^[9, 10, 11, 12, 13, 14, 15, 16, 17]^

The underlying mechanism linking OSA and rheumatic disease remains unclear. One potential explanation is that OSAinduced abnormal inflammatory states may contribute to the development of rheumatic disease.^[11]^ Additionally, some rheumatic disease may destroy the temporomandibular joint, leading to acquired retrognathia/micrognathia and occipitocervical lesions. Rheumatic disease are disease states with an excessive inflammatory response, which may lead to upper respiratory tract inflammation and collapsibility.^[18]^ Another factor could be the use of glucocorticoids to treat rheumatic disease. Excessive glucocorticoids may cause adipose tissue redistribution to the facial and neck regions, potentially exacerbating OSA.^[18]^ However, it should be noted that there is currently no direct evaluation of the potential mechanism between OSA and rheumatic disease.

Mendelian randomization (MR) design is a technique in epidemiological research that uses genetic variation as a tool variable to evaluate the potential causal relationship between exposure and outcome.^[19, 20, 21]^ The advantage of MR is that genetic variation is randomly assigned when passed on to offspring, independent of self-selection behavior, and similar to randomized clinical trials (RCTs). Therefore, this design can effectively avoid potential confounding factors or reverse causal relationships to strengthen causal reasoning.

Thus, this study mainly examines the potential causal relationship between OSA and rheumatic disease with a two-sample bidirectional MR design. We select eight rheumatic disease (*i.e*., AS, RA, SLE, gout, SS, BD, PsA, and SSc) as factors related to OSA.

## Methods

### Study Design

**Figure 1:**
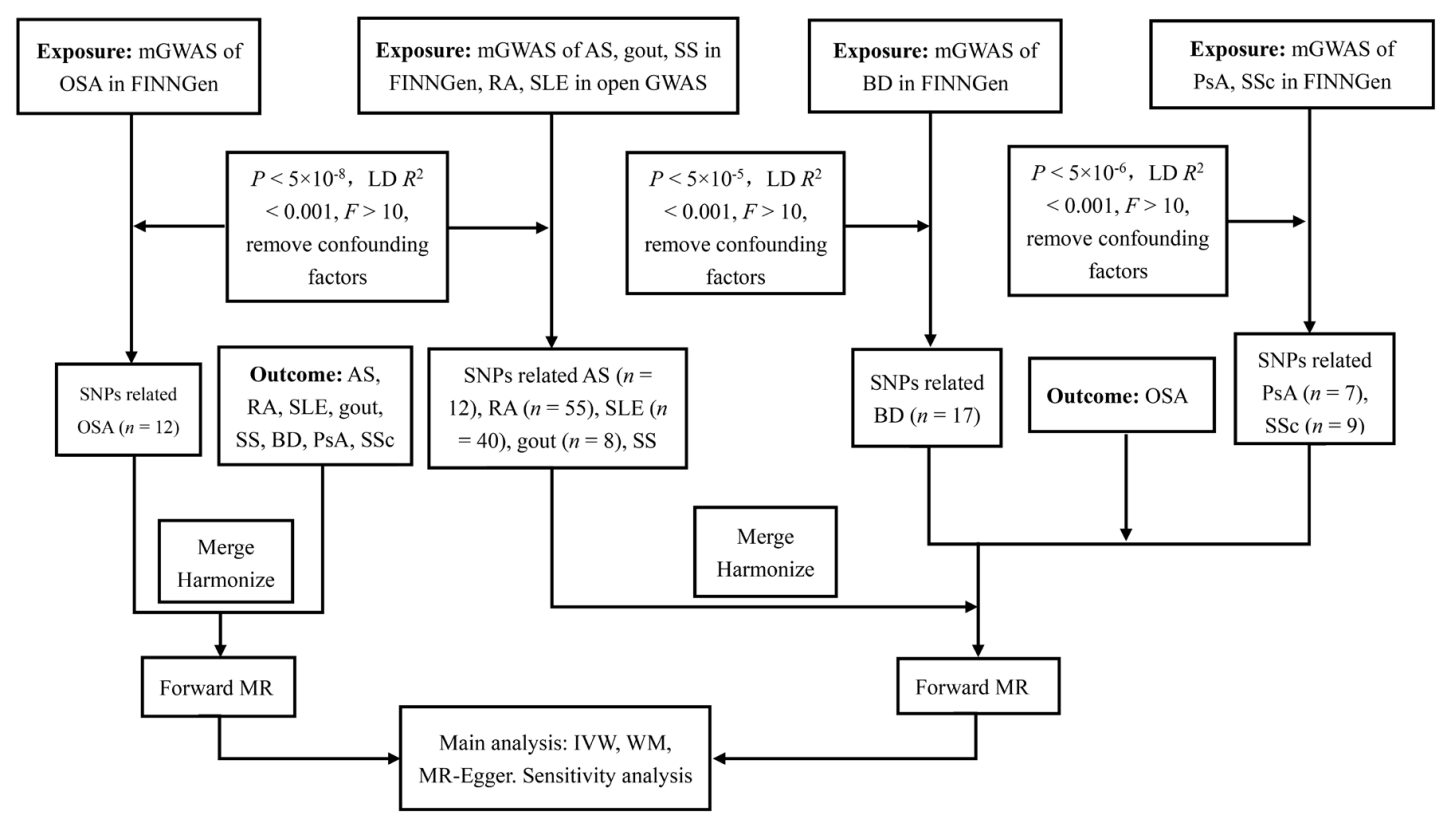
Flowchart of the causal inference between OSA and rheumatic disease. GWAS, genome-wide association studies; LD, linkage disequilibrium; SNPs, single nucleotide polymorphisms; MR, mendelian randomized; IVW, inverse variance weighted; WM, weighted median; OSA, obstructive sleep apnea; AS, ankylosing spondylitis; RA, rheumatoid arthritis; SLE, Systemic lupus erythematosus; SS, Sjogren’s syndrome; BD, Behcet disease; PsA, Psoriatic arthritis; SSc, Systemic sclerosis.

We conducted a two-sample MR analysis to explore the causal relationship between OSA and rheumatic disease, including AS, RA, SLE, gout, SS, BD, PsA, and SSc. Genetic variation as an instrumental variable (IV) needs to follow three assumptions: (1) reliably and robustly correlated with exposure, (2) independent of risk factors and confounding factors, and (3) genetic variation is only associated with outcome through selective exposure rather than through alternative pathways. This analysis utilized recently published genome-wide association studies (GWASs) based on the summary data. This study aims to explore the causal relationship between OSA and rheumatic disease through univariate bidirectional two-sample MR analysis. We evaluated whether a causal relationship exists between OSA and rheumatic disease and investigated whether there is a causal relationship between rheumatoid immune diseases and OSA. The design of our MR framework is illustrated in Figure 1.

### Data Sources

Summary statistics of OSA in the European population were obtained from FinnGen Release 9 (https://www.finngen.fi/en). These GWAS data included 38,998 OSA cases and 336,659 normal controls of European ancestry (375,657 subjects in total). Data for AS, gout, SS, BD, PsA, and SSc were extracted from the FinnGen Release 9 (https://www.finngen.fi/en). The data for SLE and RA were acquired from the Integrative Epidemiology Unit (IEU) Open GWAS database (https://gwas.mrcieu.ac.uk/). The sample sizes for each of rheumatic diseases were as follows: AS (2,860 cases and 270,964 controls), RA (14,361 cases and 42, 923 controls), SLE (5,201 cases and 9,066 controls), gout (8,489 cases and 240,862 controls), SS (2,495 cases and 365,533 controls), BD (85 cases and 365,522 controls), PsA (3,186 cases and 240,862 controls), and SSc (619 cases and 365,513 controls). Detailed information on genetic data is provided in Table 1.

**Table 1:** Description of GWAS used for OSA and rheumatic diseases

Phenotype	Cohort	No. Samples	No. cases	No. controls	SNPs	Ancestry
OSA	FG	375,657	38,998	336,659	20,170,208	European
AS	FG	273,824	2,860	270,964	20,166,920	European
RA	EBI	58,284	14,361	42,923	9,488,501	European
SLE	EBI	14,267	5,201	9,066	14,267	European
Gout	FG	249,351	8,489	240,862	20,165,508	European
SS	FG	368,028	2,495	365,533	20,170,011	European
BD	FG	172,044	78	171,966	20,169,936	European
PsA	FG	244,048	3,186	240,862	20,164,984	European
SSc	FG	366,132	619	365,513	20,169,974	European

GWAS, genome-wide association studies; SNPs, single nucleotide polymorphisms; OSA, Obstructive sleep apnea; AS, Ankylosing spondylitis; RA, Rheumatoid arthritis; SLE, Systemic lupus erythematosus; SS, Sjogren’s syndrome; BD, Behcet disease; PsA, Psoriatic arthritis; SSc, Systemic sclerosis. FG, FinnGen; EBI, European Bioinformatics Institute.

### Selection of Instrumental Variables (IVs)

In the current study, we classified single nucleotide polymorphisms (SNPs) as IVs. To fulfill three assumptions for the MR study design, firstly, we identified candidate IVs using summary statistics from GWAS with a significance threshold of *P* < 5e^-8^ for exposure-associated SNPs. In order to obtain a relatively large number of IVs for BD, we selected SNPs on a lower significant threshold (*P* < 5e^-5^). For SSc and PsA, we selected SNPs on a lower significant threshold (*P* < 5e^-6^). We applied a clumping technique, filtering SNPs with a linkage disequilibrium (LD) threshold of *R*^2^ < 0.001 and a window size of 10,000 kb to ensure independence among the IVs. Furthermore, to assess the strength of the selected IVs, we calculated the *R*^2^ and *F* statistics for each SNP using the equation: *R*^2^ = 2 × EAF × (1-EAF) ×β^2^ and *F* statistic= *R*^2^× (N-2)/(1-*R*^2^), where EAF represents the effect allele frequency, β is an estimate of the genetic effect of each SNP on the exposure, and N is the sample size of the exposure GWAS data. An *F* value greater than 10 indicates that the SNP is sufficiently robust to mitigate potential bias.^[22]^ In addition, SNPs closely associated with OSA were eliminated by searching PhenoScanner (http://www.Phenoscanner.cam.ac.uk/) and GWAS Catalog (*P* < 1e^-5^), such as obesity, fluid retention, adenotonsillar hypertrophy, smoking, and drinking. In the same way, we eliminated SNPs that may be associated with rheumatic disease, such as obesity, body mass index, smoking, and drinking. We carefully screened and removed these SNPs to ensure the integrity of the IVs. Following rigorous selection criteria, the remaining SNPs were considered eligible IVs.

Based on these rigorous criteria, 12 independent SNPs for OSA, 12 SNPs for AS, 55 SNPs for RA, 40 SNPs for SLE, 8 SNPs for gout, 6 SNPs for SS, 17 SNPs for BD, 7 SNPs for PsA and 9 SNPs for SSc were separately extracted from the summary statistics datasets (Table 2, 3).

**Table 2:** Causal effects of OSA on rheumatic disease in MR analysis in European individuals

Exposure	Outcome	SNPs (*n*)	Methods	OR (95% CI)	*P* value
OSA	AS	11 (12)	IVW (FEM)	1.0053 (0.7105, 1.4224)	0.9763
			IVW (REM)	1.0053 (0.7376, 1.3701)	0.9734
			MR Egger	0.7816 (0.2523, 2.4217)	0.6794
			Weighted median	0.9848 (0.6178, 1.5698)	0.9487
OSA	RA	11 (12)	IVW (FEM)	0.8609 (0.6971, 1.0632)	0.1642
			IVW (REM)	0.8609 (0.6720, 1.1029)	0.2359
			MR Egger	0.5153 (0.1723, 1.5411)	0.2659
			Weighted median	1.0000 (0.7188, 1.3912)	1.0000
OSA	SLE	8 (12)	IVW (FEM)	1.2363 (0.7475, 2.0448)	0.4087
			IVW (REM)	1.2363 (0.8639, 1.7691)	0.2460
			MR Egger	1.0543 (0.1427, 7.7863)	0.9604
			Weighted median	1.2129 (0.6592, 2.2316)	0.5349
OSA	Gout	11 (12)	IVW (FEM)	1.1559 (0.9390, 1.4230)	0.1719
			IVW (REM)	1.1559 (0.8804, 1.5176)	0.2968
			MR Egger	1.6126 (0.6532, 3.9814)	0.3271
			Weighted median	0.9881 (0.7226, 1.3510)	0.9402
OSA	SS	11 (12)	IVW (FEM)	1.2603 (0.8702, 1.8252)	0.2208
			IVW (REM)	1.2603 (0.8295, 1.9148)	0.2784
			MR Egger	1.5525 (0.3706, 6.5038)	0.5621
			Weighted median	1.4250 (0.8336, 2.4359)	0.1954
OSA	BD	11 (12)	IVW (FEM)	2.5129 (0.3467, 18.2147)	0.3619
			IVW (REM)	2.5129 (0.2216, 28.4961)	0.4570
			MR Egger	1.5632 (0.0004, 6452.3152)	0.9186
			Weighted median	2.1278 (0.1068, 42.3824)	0.6208
OSA	PsA	11 (12)	IVW (FEM)	0.8277 (0.5495, 1.2468)	0.3657
			IVW (REM)	0.8277 (0.5259, 1.3028)	0.4139
			MR Egger	0.9628 (0.2038, 4.5491)	0.9629
			Weighted median	1.2070 (0.4017, 3.6261)	0.8454
OSA	SSc	11 (12)	IVW (FEM)	1.0209 (0.4894, 2.1293)	0.9561
			IVW (REM)	1.0209 (0.5566, 1.8723)	0.9468
			MR Egger	0.3383 (0.0311, 3.6833)	0.3968
			Weighted median	0.9290 (0.3529, 2.4455)	0.8815

MR, mendelian randomized; SNPs, single nucleotide polymorphisms; OR, odds ratio; CI, confidence interval; IVW, inverse variance weighted; FEM, fixed effects model; REM, random effects model. OSA, Obstructive sleep apnea; AS, Ankylosing spondylitis; RA, Rheumatoid arthritis; SLE, Systemic lupus erythematosus; SS, Sjogren’s syndrome; BD, Behcet disease; PsA, Psoriatic arthritis; SSc, Systemic sclerosis.

**Table 3:** Causal effects of rheumatic disease on OSA in MR analysis

Exposure	Outcome	SNPs (*n*)	Method	OR (95% CI)	*P* value
AS	OSA	10 (12)	IVW (FEM)	1.0239 (1.0086, 1.0394)	0.0021
			IVW (REM)	1.0239 (1.0072, 1.0407)	0.0047
			MR Egger	1.0374 (1.0089, 1.0668)	0.0326
			Weighted median	1.0287 (1.0109, 1.0467)	0.0014
RA	OSA	47 (55)	IVW (FEM)	0.9995 (0.9826, 1.0168)	0.9558
			IVW (REM)	0.9995 (0.9802, 1.0192)	0.9614
			MR Egger	0.9870 (0.9472, 1.0284)	0.5350
			Weighted median	1.0063 (0.9783, 1.0351)	0.4106
SLE	OSA	34 (40)	IVW (FEM)	0.9977 (0.9880, 1.0076)	0.6476
			IVW (REM)	0.9977 (0.9869, 1.0086)	0.6809
			MR Egger	0.9853 (0.9612, 1.0100)	0.2488
			Weighted median	1.0008 (0.9853, 1.0165)	0.9201
			Weighted median	1.0063 (0.9783, 1.0351)	0.4106
Gout	OSA	4 (8)	IVW (FEM)	0.9796 (0.9186, 1.0447)	0.5299
			IVW (REM)	0.9796 (0.9141, 1.0497)	0.5591
			MR Egger	1.0572 (0.9383, 1.1912)	0.4572
			Weighted median	0.9961 (0.9240, 1.0497)	0.9178
SS	OSA	4 (6)	IVW (FEM)	1.0039 (0.9660, 1.0432)	0.8431
			IVW (REM)	1.0039 (0.9710, 1.0379)	0.8191
			MR Egger	0.9938 (0.8463, 1.1669)	0.9460
			Weighted median	1.0041 (0.9586, 1.0518)	0.8622
BD	OSA	14 (17)	IVW (FEM)	0.9982 (0.9881, 1.0083)	0.7210
			IVW (REM)	0.9982 (0.9850, 1.0114)	0.7838
			MR Egger	1.0127 (0.9814, 1.0451)	0.4459
			Weighted median	1.0043 (0.9884, 1.0204)	0.5973
PsA	OSA	6 (7)	IVW (REM)	1.0348 (0.9820, 1.0905)	0.2005
			IVW (FEM)	1.0348 (1.0020, 1.0687)	0.0372
			MR Egger	1.0243 (0.9408, 1.1151)	0.6093
			Weighted median	1.0323 (0.9865, 1.0801)	0.1696
SSc	OSA	8 (9)	IVW (FEM)	0.9842 (0.9683, 1.0003)	0.0544
			IVW (REM)	0.9842 (0.9755, 0.9929)	0.0004
			MR Egger	0.9994 (0.9641, 1.0360)	0.9748
			Weighted median	0.9863 (0.9662, 1.0069)	0.1908

MR, mendelian randomized; SNPs, single nucleotide polymorphisms; OR, odds ratio; CI, confidence interval; IVW, inverse variance weighted; FEM, fixed effects model; REM, random effects model. OSA, Obstructive sleep apnea; AS, Ankylosing spondylitis; RA, Rheumatoid arthritis; SLE, Systemic lupus erythematosus; SS, Sjogren’s syndrome; BD, Behcet disease; PsA, Psoriatic arthritis; SSc, Systemic sclerosis.

### Two-sample MR Analysis

In the univariable MR analysis, the traditional method used was the inverse variance weighted (IVW) approach. This method combines the Wald ratio estimates for each instrumental SNP through a model similar to meta-analysis, providing an overall estimate of the effect.^[19]^ When there was no heterogeneity (*P* > 0.05), the fixed effects model (FEM) was used; if heterogeneity existed (*P* < 0.05), the multiplicative random-effect model (REM) was used. However, the IVW method only generates an unbiased estimate under the specific assumptions that there is no invalid instrument and horizontal pleiotropy. Several sensitivity analysis methods were employed to validate the robustness of the effect estimate obtained from the IVW method, including the weighted median (WM) and MR-Egger. The WM validly estimates the causal effect even if up to half of the weights are driven by invalid instruments.^[23]^ The intercept of MR-Egger regression captures the average pleiotropic effect across the genetic variants, where a significant non-zero intercept term can indicate directional pleiotropy.^[24]^

### Sensitivity Analysis

Cochrane’s Q value was applied to assess the heterogeneity between SNPs. Visual funnel plots were applied to evaluate publication bias and directional pleiotropy by examining symmetry.^[25]^ Horizontal pleiotropy was detected *via* conducting the MR-Egger-intercept and the MR pleiotropy residual sum and outlier (MR-PRESSO) tests. The outlier test would compare the expected and observed distribution of each variant to identify outlier variants. If any outliers were detected, they would be eliminated, and the corrected results would be re-evaluated.^[26]^ In addition, a leave-one-out (LOO) analysis was applied to avoid horizontal pleiotropy induced by a single SNP, which systematically eliminated the SNP and calculated the effect of the remaining SNPs.^[27]^ All statistical analyses were conducted using the “TwoSampleMR” package (version 0.5.7) and the “MRPRESS” package (version 1.0) in R software (version 4.3.1).

## Results

### Causal Effects of OSA on Rheumatic Diseases

As mentioned above, we got 12 independent SNPs from OSA through rigorous criteria. Table S3 displays statistics for the 12 SNPs chosen as valid IVs in this MR analysis. The general and each IV’s *F* values were greater than the empirical threshold 10, suggesting weak instrumental factors did not cause significant bias. Then, we harmonized the summary statistics of exposure and outcome by aligning the efects to the identical reference alleles and removed abnormal, ambiguous, and palindromic SNPs from IVs. Finally, SNPs related to OSA screened in the rheumatic immune disease database were 8 SNPs in SLE and 11 SNPs in other rheumatic disease (Table 2).

**Figure 2:**
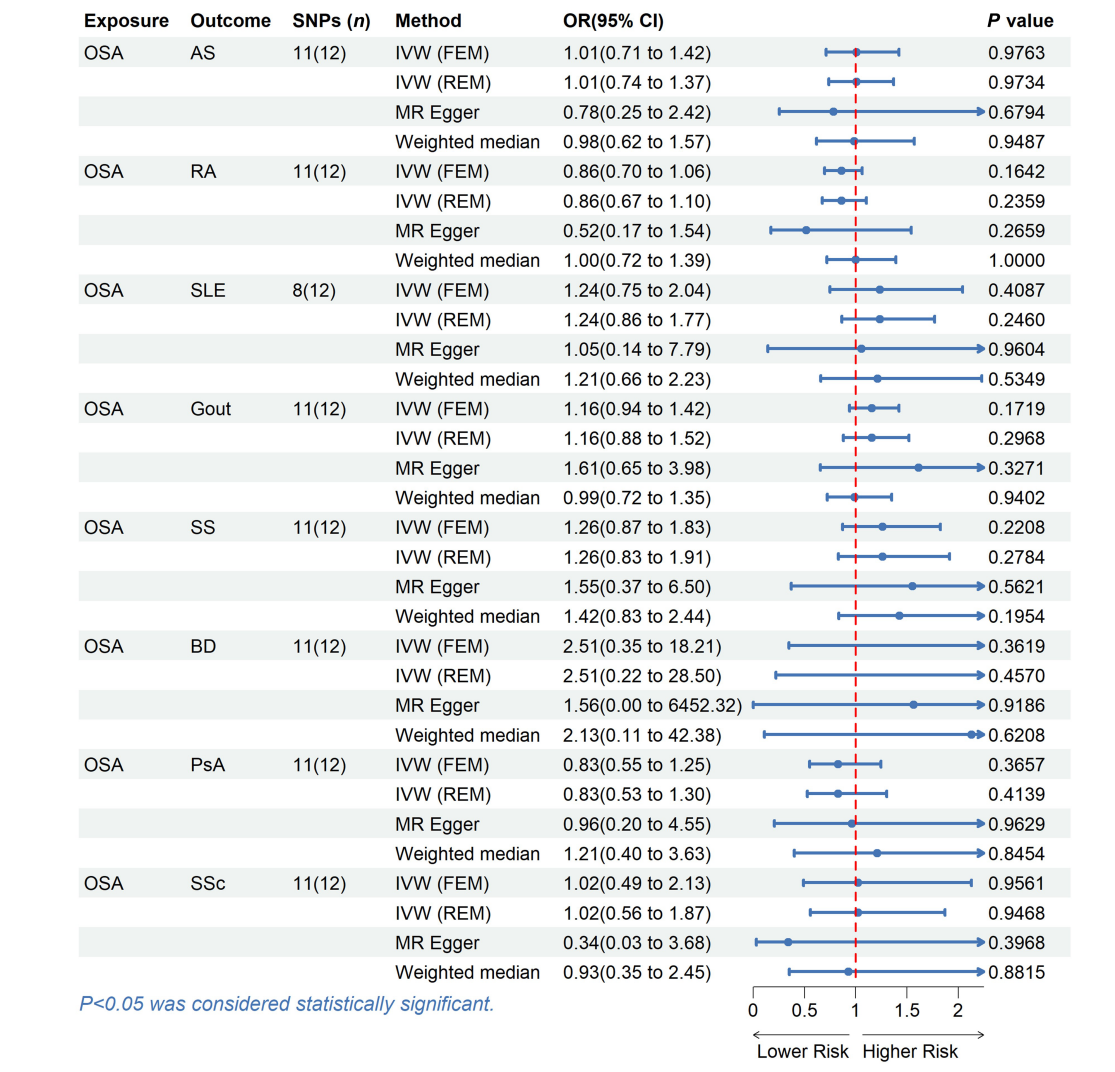
MR estimates for the causal effect of OSA on rheumatic disease. SNPs, single nucleotide polymorphisms; OR, odds ratio; CI, confidence interval; IVW, inverse variance weighted; FEM, fixed effects model; REM, random effects model; OSA, Obstructive sleep apnea; AS, Ankylosing spondylitis; RA, Rheumatoid arthritis; SLE, Systemic lupus erythematosus; SS, Sjogren’s syndrome; BD, Behcet disease; PsA, Psoriatic arthritis; SSc, Systemic sclerosis.

There was no evidence supporting causal relationships between OSA and rheumatic disease. Using the IVW method, the MR results indicated that OSA could not directly affect the risk of rheumatic disease (all *P* > 0.05). These findings were confirmed using other MR methods, including WM and MR Egger (Table 2, Figure 2).

The Cochran’s Q statistic (*P* > 0.05), MR Egger intercept test (*P* > 0.05), and MRPRESSO (Global *P* > 0.05) failed to identify any directional pleiotropy or heterogeneity (Table S1). Besides, the forest plots, scatter plots, funnel plots, and LOO plots are displayed in Figure S1–4.

### Causal Effects of Rheumatic Diseases on OSA

**Figure 3:**
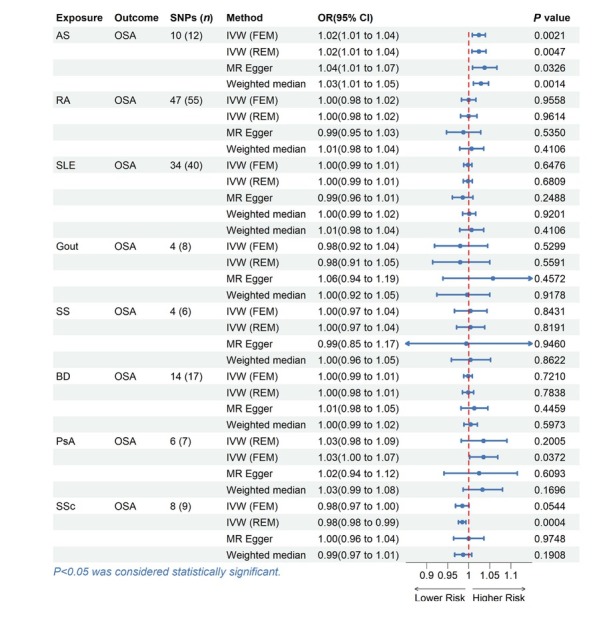
MR estimates for the causal effect of rheumatic disease on OSA. SNPs, single nucleotide polymorphisms; OR, odds ratio; CI, confidence interval; IVW, inverse variance weighted; FEM, fixed effects model; REM, random effects model; OSA, Obstructive sleep apnea; AS, Ankylosing spondylitis; RA, Rheumatoid arthritis; SLE, Systemic lupus erythematosus; SS, Sjogren’s syndrome; BD, Behcet disease; PsA, Psoriatic arthritis; SSc, Systemic sclerosis.

We conducted a reverse analysis to assess further the causal effect of rheumatic disease on OSA. After removing abnormal, ambiguous, and palindromic SNPs, we got 6 SNPs for PsA, 4 SNPs for gout, 4 SNPs for SS, 14 SNPs for BD, 8 SNPs for SSc, 48 SNPs for RA, 36 SNPs for SLE and 10 SNPs for AS. The F statistic value at each chosen IV was greater than 10, indicating that the chosen SNPs were adequately robust and that weak IVs were not likely to alter the causal estimate. The specific information can be found in the Table S6–13. Genetically predicted AS was associated with the risk of OSA (IVW: OR = 1.0239, 95% CI = 1.0086 to 1.0394, *P* = 0.0021; MR-Egger: OR = 1.0374, 95% CI = 1.0089 to 1.0668, *P* = 0.0326; weighted median: OR = 1.0287, 95% CI = 1.0109 to 1.0467, *P* = 0.0014). However, no evidence suggests a causal relationship between OSA and other rheumatic disease (Table 3, Figure 3).

Except for the heterogeneity of PsA, our analysis did not uncover any signs of heterogeneity or pleiotropy in the causal relationship between rheumatic disease and OSA, as assessed by Cochrane’s Q (*P* > 0.05) and MR Egger intercept (*P* > 0.05) (Table S2). Through MR-PRESSO and LOO analysis, one outlier (rs6679677) was identified in RA, and two outliers (rs6679677, rs28361029) were identified in SLE, which indicated that the IVs of exposure and outcome have significant horizontal pleiotropy. After removing outliers, the calculation was repeated. It was found that there was no horizontal pleiotropy, and these results were not significantly affected. Figure S5–8 contains displays of some plots, including forest plots, scatter plots, funnel plots, and LOO plots.

## Discussion

In this bidirectional MR study, we investigated the causal relationship between OSA and rheumatic disease and identified genetic variations representing this impact using publicly accessible large-scale GWAS data. Our results showed that only gene-predicted AS had a causal relationship with OSA, with no evidence supporting a causal link between OSA and other rheumatic disease. Sensitivity analysis using several MR models indicated that the conclusion was reliable. To our knowledge, this is the first MR study that investigated the bidirectional causal relationship between OSA and rheumatic disease.

In the past few decades, a wealth of observational evidence 48 has linked OSA to rheumatic disease. For example, Chen *et al*. conducted a retrospective cohort study in Taiwan, finding that OSA patients, with no prior autoimmune diseases, had a significantly higher risk of developing RA, SS, and BD compared to controls.^[6]^ A meta-analysis involving 18 studies suggested that OSA may be a potential risk factor for the development of hyperuricemia and gout.^[28]^ A prospective cohort study shows that people with OSA have approximately twice the risk of developing psoriasis or PsA than the general population.^[8]^ In addition, a study based on polysomnography (PSG) showed that OSA is more common in RA patients than in the general population.^[10]^ And a cross-sectional study found an increased prevalence of OSA among patients with SLE compared to healthy controls.^[12]^ Moreover, a retrospective study demonstrated that the overall risk of OSA was significantly higher in SS and BD patients compared to controls.^[11]^ In a prospective study, 22 (58%) of 38 SSc patients developed OSA.^[15]^

Through MR analysis, we found a statistically significant increase in the risk of OSA in AS patients. The underlying mechanism for this association remains unclear. AS is a chronic inflammatory rheumatic disease with the spine as the primary lesion site, affecting the sacroiliac joint, causing spinal rigidity and fibrosis, resulting in structural and functional impairment, as well as decreased quality of life.^[29]^ The global prevalence of AS ranges from 0.07% to 0.32%.^[30]^ The mechanism of OSA in AS patients can be analyzed from the following two aspects. On the one hand, when the lesions affect the cervical spine, there may be spasms and atrophy of the neck muscles, cervical deformities, rigidity, and limited mobility, which lead to narrowing of the pharyngeal airway gap,^[31]^ causing obstruction, resulting in respiratory pauses and low ventilation.^[32]^ On the other hand, patients with AS have elevated levels of inflammatory cytokines, and their systemic inflammatory response may lead to local swelling and edema of the upper airway soft tissue, resulting in airway stenosis.^[33]^ Clinical research supports the causal relationship between AS and OSA. For instance, a national cohort study showed that AS patients had an increased risk of developing OSA compared to controls.^[17]^ Walsh *et al*. examined the co-morbidity burden in patients with AS, and discovered that the prevalence of OSA was higher in AS patients compared to the control group (8.8% *vs*. 5.1%, *P* < 0.001).^[34]^ However, our MR analysis failed to discover other associations. Because of the strict criteria for selecting IVs, our MR study may not be able to bring a slight increase in risk due to limited statistical power. Therefore, we cannot completely deny the possible causal relationship between OSA and rheumatic disease.

The advantage of this study is that, firstly, SNPs are randomly assigned during inheritance and independent of confounding factors. The bias caused by reverse causality or confounding in MR design is greatly reduced. Secondly, the data came from large-scale GWAS with a large sample size to obtain statistically reliable power. Furthermore, this study significantly reduced potential biases caused by population stratification by limiting the data to participants of European ancestry.

Some limitations must be acknowledged in this study. First, although limiting the sample to individuals with European ancestry can reduce demographic bias, it may make our findings more difficult to apply to other populations. Second, as this study used aggregated data, further stratified data analysis based on individual-level data is needed to examine the causal relationship between OSA and rheumatic disease. In addition, we did not conduct multivariate and mediation MR analyses to distinguish between indirect and direct causal relationships. Finally, this study did not differentiate the distribution and subtypes of various severity levels of OSA. Future work should reveal the potential biological mechanisms between them through multivariate and mediation analysis.

## Conclusions

Current research has found a statistically significant increase in the risk of OSA in AS patients. However, no bidirectional causal association was found between other rheumatic disease and OSA. The direct mechanism of the causal relationship between OSA and rheumatic disease needs further research.
